# Microalgal Cell Wall Dynamics at Different Growth Stages Under Cold Stress Conditions: A Study of Ultrastructure and Chemical Composition

**DOI:** 10.1111/ppl.70960

**Published:** 2026-06-04

**Authors:** María González‐Hourcade, Francesco G. Gentili, Dinesh Fernando

**Affiliations:** ^1^ Department of Forest Bioeconomy and Technology Swedish University of Agricultural Sciences Umeå Sweden; ^2^ Department of Forest Bioeconomy and Technology Swedish University of Agricultural Sciences Uppsala Sweden

## Abstract

Microalgae have been gaining attention for biotechnological purposes in different fields, such as food, energy, cosmetics, and bioremediation. However, due to habitat variation across the planet, there is still a scarcity of information on microalgae development in extreme environments, such as those with low temperatures. The aim of this study was to investigate the effects of growth stage and cold stress (5°C) on cell wall features, including ultrastructure, and main polysaccharides in three microalgal species from Northern Sweden: *Coelastrella* sp. 3–4, *Chlorella vulgaris* sp. 13–1, and *Scenedesmus* sp. B2‐2. All microalgal strains grown at 25°C showed increased cell wall thickness between the exponential and stationary phases by around 45%, with *Scenedesmus* showing an increase of up to 230%. Under cold stress, the cell wall thickness between the exponential and stationary phases had the greatest increase of 68% in *Scenedesmus*. However, both *Chlorella vulgaris* sp. 13–1 and *Scenedesmus* sp. B2‐2 showed that cold stress stimulated the formation of a thicker cell wall during exponential growth compared to control growth conditions. The layer configuration showed a differentiation, with *Chlorella* presenting a bilayer cell wall and the cell walls of *Coelastrella* and *Scenedesmus* being mainly composed of several layers distinguishable by transmission electron microscopy. Cold stress altered the microalgal cell wall ultrastructure and morphology. Glycosyl‐linkage analysis did not show a change in the composition of the main cell wall polysaccharides under cold stress.

## 1 Introduction

Microalgae are photosynthetic microorganisms that convert sunlight and CO_2_ into carbon‐rich compounds, making them valuable for biotechnological applications, such as biofuels (Lage et al. 2018), food, feed (Bernaerts et al. 2018), nutraceuticals, and pollutant removal from wastewater (Gentili and Fick 2017; Lage et al. 2018; González‐Hourcade et al. 2022). Due to their fast growth rates, the versatility of the compounds they produce, especially biologically valuable products, and their ability to adjust their biochemical composition depending on cultivation conditions (Dolganyuk et al. 2020; González‐Hourcade et al. 2023), microalgae have received increasing interest in the last decade as an important sustainable resource for the biotechnology industry.

These autotrophic microorganisms are found in a range of aquatic environments, including freshwater, marine, and extreme environments, such as deserts (Perera et al. 2018) to polar (Chekanov et al. 2019) and subarctic areas (Ferro, Gentili, and Funk 2018; Goecke et al. 2020). Some microalgae grow at high latitudes characterised by extremely low temperatures and complete darkness in winter, as well as continuous light and high UV in the summer (Kumari et al. 2022). Temperature and light are the main factors for microalgal cultivation. Similar to other stress factors, cold exposure stimulates microalgae to produce value‐added products, such as proteins, lipids, pigments, and carbohydrates (Markou and Nerantzis 2013; Wan et al. 2019; Liyanaarachchi et al. 2021; Lindberg et al. 2022), but may also affect the structural integrity and physiological functions.

One of the most challenging steps of downstream processes involving microalgal biomass is cell wall (CW) disruption (Baudelet et al. 2017). The CW is a crucial component of the cell that affects the physiology of microalgae, providing a physical and mechanical barrier that protects cells from the surrounding environment. The composition and structure/configuration of the CW present a variety between species (Finkel et al. 2016; Baudelet et al. 2017). The growth and survival of microalgae are often limited by environmental stressors, which modify the architecture and biochemical composition of their CWs, affecting their structural integrity and physiological functions. For example, Rashidi et al. (2019) showed that the monosaccharide composition of *Neochloris oleoabundans* differs depending on whether fresh‐ or salt‐water is used in the cultivation medium. Moreover, changes in CW thickness (increase or decrease) as a result of different stress conditions have been previously shown (Donk et al. 1997; Karsten and Holzinger 2011). Munns et al. (1983) described a thicker CW of up to 70% in *Chlorella emersonii* when grown in a hypersaline culture, suggesting that the CW thickness varies with growth condition changes. Long‐term nitrogen deficiency induced a thicker CW in *Vischeria* sp. WL1, increasing the CW rigidity and desiccation resistance (Liang et al. 2025). Few studies have focused on the potential of microalgae grown under cold stress (Artamonova et al. 2017; Hulatt et al. 2017; Ferro, Gorzsás, et al. 2018) compared to studies on other abiotic stressors.

Ferro, Gentili, and Funk (2018) isolated 62 eukaryotic microalgae from Northern Sweden from fresh and wastewater sources, and *Scenedesmus*, *Desmodesmus*, *Chlorella*, and *Coelastrella* were the dominant species in these habitats. Our recent study showed a detail characterisation of some unicellular microalgal species, including *Scenedesmus*, *Chlorella*, *Haematococcus*, and *Coelastrella* from Northern Sweden, revealing their morphological organisation with an emphasis on major CW components and their micro‐distribution (González‐Hourcade et al. 2023). These microalgal species were found to express phenotypic plasticity when exposed to cold stress; consequently, they adapted to the change in the environment by modifying their cellular morphology/organisation and major CW components. For example, microalgae showed micro‐morphological changes, such as cell size and shape, cell organisation, and CW ornamentation, with *Scenedesmus* sp. B2–2 changing from a four‐celled coenobium to a unicellular organisation containing multi‐spined CWs at low temperatures. Modifications in the presence/absence and micro‐distribution of CW biochemical components, such as pectin, algaenan, and other exopolysaccharides, were observed. A previous study has shown that *Scenedesmus* sp. B2–2 increases the CW thickness over time at 25°C but especially at 5°C (Spain et al. 2024). Both *Scenedesmus* sp. B2–2 and *Chlorella vulgaris* 13–1 have been previously isolated in a subarctic region and grew at 5°C (Ferro, Gorzsás, et al. 2018; González‐Hourcade et al. 2023).

The sugar composition of the CW of microalgae belonging to the genus *Chlorella* varies greatly, mainly including galactose and rhamnose, but in some strains, it may also contain arabinose and glucose (Baudelet et al. 2017). Glucose is the main sugar in the CW of *C. vulgaris* (Demir‐Yilmaz et al. 2023). *Scenedesmus* spp. showed mainly mannose and glucose in its CW (Baudelet et al. 2017). A microalgal, *Trachydiscus guangdongensis*, belonging to the class of Eustigmatophyceae, had a CW composition composed mainly (66%) of galactose (Gao et al. 2019). There is still a lack of knowledge concerning the ultrastructural features and related biochemical/CW polysaccharide changes in microalgal CWs in response to cold temperature exposure.

Therefore, the aim of the present study was to investigate the ultrastructural organisation and main polysaccharide composition, including structural connections between sugar units, of the CWs of three microalgal species isolated in subarctic locations: *C. vulgaris* 13–1, *Scenedesmus* sp. B2‐2, and *Coelastrella* sp. 3–4 (Ferro, Gentili, and Funk 2018). Emphasis was given to the primary features of the CW (e.g., ultrastructure, including CW appendages/outgrowth, thickness, layer configuration, and biochemical composition) during the growth of microalgal cells, which were investigated using transmission electron microscopy (TEM) and chemical analysis. The study was carried out by examining two crucial phases of the growth curve, namely the exponential and stationary phases, under normal temperature (25°C) and cold stress conditions (5°C). Species‐specific strategies of stress adaptation were identified. The results of the present study improve our understanding of how cold‐tolerant microalgal strains adapt their CWs in response to low temperature conditions, offering an ecological perspective on their ability to regulate their CW architecture to support survival and growth under changing climate conditions. From a biotechnological standpoint, an increase in CW thickness may have implications for downstream processes involving efficient CW disruption.

## 2 Material and Methods

### 2.1 Microalgal Cultivation and Cold Tolerance Experiments

The microalgal strains used in this study were previously isolated and genetically characterised by Ferro, Gentili, and Funk (2018). The species used were *Coelastrella* sp. 3–4, *C. vulgaris* sp. 13–1, and *Scenedesmus* sp. B2‐2, and details of the spp. and their growing conditions have been reported previously (González‐Hourcade et al. 2023). In brief, microalgal cultures were cultivated in 1‐L transparent glass bottles containing Bold's basal medium at pH 6.5. The algae were grown in a climate chamber (Conviron A1000; Controlled Environments Limited). The three strains in culture bottles were grown under control (25°C, 18/6 h light/dark at 130 μmol m^−2^ s^−1^) and cold stress conditions (5°C, 18/6 h light/dark at 130 μmol m^−2^ s^−1^) under agitation at 120 rpm with a bubbling rate of 0.1–0.2 L air/min per L of liquid culture. Each microalgal strain was cultivated in triplicate.

To monitor the growth curve, the optical density (OD) of the culture was measured at 750 nm every day. At the start of the cultivation, the OD of the culture was 0.047. The microalgae were harvested during the exponential growth (12 days) and stationary phases (21 days) under control conditions, as previously described by González‐Hourcade et al. (2023). Cultures grown under cold stress were harvested at the exponential and stationary phases after 40 and 55 days, respectively. In the case of *Coelastrella* sp. 3–4, the biomass culture under cold stress was not enough for the experimental methodology; therefore, it was prioritised for glycosyl‐linkage analysis. The microalgae biomass was placed into 50‐mL plastic tubes and centrifuged at 4000 × g for 5 min. Microalgae pellets were then re‐suspended 3 times in 50 mL of MiliQ water, flash‐frozen in liquid nitrogen, and stored frozen at −80°C until analysis.

### 2.2 Preparation of Whole Cell Wall Samples

Approximately 1–1.5 g of cell pellets were milled with a mortar and pestle with liquid nitrogen until a fine powder was obtained. The powder was suspended and pre‐washed in 80% ethanol, centrifuged, and re‐suspended in ethanol overnight under gentle agitation at room temperature. They were then washed several times for 15 min each, first with 80% and 100% ethanol and then with n‐hexane: acetone (1:1) to remove most of the organic compounds, such as fatty acids, proteins, and pigments. Final washes were performed with 100% n‐hexane and left to dry under a fume hood for 24 h. The alcohol‐insoluble resistant materials were collected and weighed. Following this protocol, centrifugation was performed at 5000 *g* for 5 min.

### 2.3 Transmission Electron Microscopy (TEM)

Microalgae cells were incubated using a fixative solution of 3% (v/v) glutaraldehyde and 2% (v/v) paraformaldehyde in 0.1 M cacodylate buffer (pH 7.2) for 24 h at room temperature under continuous rotational agitation. After 3 washes in pure buffer, samples were post‐fixed in 1% (w/v) aqueous osmium tetroxide at room temperature for 1 h. After washing (3 times in pure buffer), samples were dehydrated in ethanol (50, 70, 90, and 100%) at room temperature for 10 min in each solution. Samples were embedded in Spurrs resin (Spurr 1969) and polymerised at 70°C for 72 h. Ultrathin sections (70–90 nm) were cut using a Reichert Ultracut E ultramicrotome, mounted on 200‐mesh copper grids, and post‐stained with 2% (w/v) aqueous uranyl acetate for 30 min. Samples were observed and corresponding TEM micrographs were acquired with a Talos L120C transmission electron microscope operating at 120 kV, equipped with a Ceta CEMOS detector, 4 × 4 k pixels, operated with Velox software (all from Thermo Fisher Scientific, former FEI, Eindhoven), or a Philips CM12 TEM microscope (Philips, Eindhoven) operated at 60–80 kV.

### 2.4 Glycosyl Linkage Analysis

Glycosyl linkage analysis was performed at the Complex Carbohydrate Research Center, University of Georgia (Athens, Georgia, USA) using combined gas chromatography–mass spectrometry (GC–MS) of the partially methylated alditol acetate (PMAA) derivatives produced from the CW samples according to the method described by Anumula and Taylor (1992).

Briefly, permethylation of the CW samples prepared as described above was performed via four rounds of treatment with sodium hydroxide (15 min) and methyl iodide (30 min). The samples were hydrolysed using 2 M trifluoroacetic acid (TFA) (2 h in a sealed tube at 120°C), reduced with sodium borohydride (NaBH_4_), and acetylated using acetic anhydride/TFA. The resulting partially methylated alditol acetate (PMAAs) were analysed on an Agilent 7890A GC interfaced to a 5975C mass selective detector (electron impact ionisation mode); separation was performed on a 30‐m Supelco SP‐2331 bonded phase fused silica capillary column for the neutral residues and an EC‐1 column for the amino‐containing residue.

## 3 Results

### 3.1 Changes in Cell Wall Thickness During the Cell Cycle and Different Temperatures in Three Sub‐Arctic Microalgal spp.

**FIGURE 1:**
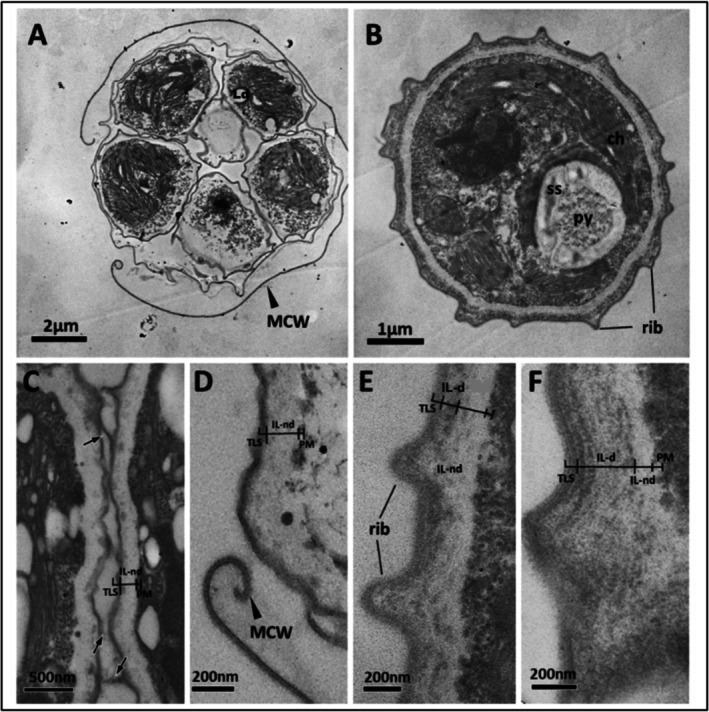
TEM micrographs of *Coelastrella* sp. 3–4 at 25°C showing cell wall (CW) development during the life cycle starting from the autosporagium to vegetative cells: (A) *Coelastrella* sp. 3–4 daughter cells in an autosporagium with an outer mother cell wall (MCW); (B) Cross‐section of a vegetative cell with cellular organelles, such as a chloroplast (ch), pyrenoids (py), and starch sheath (ss); (C) Cross‐section of two adjacent daughter cells (part of the CWs of the two cells) in an autosporagium showing details in the layered structure with an outer trilaminar sheath (TLS) layer, thick non‐electron‐dense inner layer (IL‐nd), and plasmalemma membrane (PM). Black arrows indicate thread‐like adhesion connections between two adjacent daughter cells; (D) Cross‐section of a released daughter cell with its MCW; (E, F) Cross‐section of mature CWs illustrating details. Note the newly developing electron‐dense inner layer (IL‐d; E), which was significantly thickened at a later growth stage, making the non‐electron‐dense inner layer (IL‐nd; now become innermost) relatively thin (F). More defined cell wall outgrowths existed as ribs (rib; E, F), which were thicker at the region joining the TLS of the CW and narrower at the end exposed to the outer environment.

TEM was employed to examine CW ultrastructural features of the microalgal species. Data on the CW thickness obtained from the TEM micrographs are presented in Table 1. TEM data revealed a peculiar feature in *Coelastrella* spp., whereby ribs were observed as part of the CW, extending over the CW extracellularly (Figure 1). To avoid the influence of these CW outgrowths, CW thickness measurements were collected in different areas, considering differences in the thickness between rib presence and absence zones. CW zones lacking ribs displayed thicknesses of ca 176 and 282 nm during exponential and stationary phases, respectively (Table 1). In contrast, zones containing ribs showed thickness measurements of ca 258 and 506 nm (additional data not shown in the Table) during exponential and stationary phases, respectively. The ribs were oval in shape and were of an unequal thickness from top to bottom, with the latter part thicker than the former. Thus, our observations indicate that the ribs created an extra thickening effect of ca 80 and 200 nm during the early and late stages of the cell cycle, respectively.

**TABLE 1:** Thickness of the cell wall of microalgal species at different growth stages and two temperature conditions: 25°C as control and 5°C for cold conditions (*n* = 25).

Microalgae strain	Average cell wall thickness (nm)		
	Condition	Exponential	Stationary
*Coelastrella* sp. 3–4	Control	176.29 ± 20.8^a^	281.8 ± 57.8^b^
	Cold	—	—
*Chlorella vulgaris* sp. 13–1	Control	152.07 ± 33.48^aA^	220.05 ± 30.94^bA^
	Cold	229.09 ± 6^aB^	248.98 ± 43.5^bB^
*Scenedesmus* sp. B2‐2	Control	47.27 ± 9.28^aA^	155.51 ± 16.03^bA^
	Cold	86.98 ± 13.07^aB^	146.46 ± 16.69^bA^

*Note:* Lowercase letters indicate differences between growth stages for a given strain within a row. Uppercase letters indicate differences between temperature treatments for a given strain within a column. Different letters indicate statistically significant differences (*p* < 0.05).

Under control conditions, the average CW thickness of *C. vulgaris* sp. 13–1 showed a thickness of 152 nm during the exponential growth phase, increasing to 220 nm during the stationary phase (Table 1). As is well known, the changing CW thickness was also observed as an adaptation mechanism in *C. vulgaris* sp. 13–1 under cold stress. In this study, the CW thickness was ca 229 and 249 nm during the exponential and stationary phases, respectively (Table 1). This represents an increase in the thickness of ca 50 (exponential) and 12% (stationary) compared to that of normal growth conditions, indicating major variation occurring under cold stress during the exponential phase of the cell cycle.

*Scenedesmus* sp. B2‐2 underwent an increase in CW thickness during the cell cycle under control conditions compared to the other two microalgal strains studied (Table 1). Initially, during the early stages, the CW was rather thin, measuring ca 47.3 nm. However, as the cell progressed through the cell cycle, the CW thickness significantly increased (ca 230%), reaching a thickness of 155 nm (Table 1). *Scenedesmus* sp. under low temperature conditions showed significant CW thickening compared to that under control conditions during the exponential growth phase (i.e., close to a two‐fold increase; Table 1). However, during the stationary phase, the CW thickness was slightly thicker under control conditions than that under cold stress (155.51 and 146.46 nm, respectively) (Table 1). Nonetheless, there was a CW thickness increment of ca 68% from the exponential to the stationary phase under cold stress, which was much less than the increment observed under normal growth conditions. In summary, we observed an increase in the CE thickness in all three microalgal strains as the growth phase changed from exponential to stationary.

### 3.2 Investigation of the Cell Wall Architecture During the Cell Cycle and Different Temperatures Using TEM

The CW ultrastructure and composition are poorly documented in *Coelastrella* spp. In the present study, ribs were observed as extracellular structures protruding from the CW in *Coelastrella* sp. 3–4 (Figure 1). TEM observation showed differences in the CW ultrastructure of *Coelastrella* sp. during cellular development in the exponential and stationary phases (Figure 1A,B, respectively). Detailed investigation of the cells revealed that daughter CWs in the exponential phase were composed of an electron‐dense trilaminar sheath‐type (TLS), thin outer layer and an inner thick non‐electron‐dense layer (IL‐nd) continuing up to the plasma membrane (PM) (Figure 1C,D). An outer mother cell wall (MCW) encapsulating daughter cells in an autosporangium was also observed as a TLS‐type layer consisting of a relatively electron‐lucent middle layer surrounded by outer and inner electron‐dense layers (MCW; Figure 1D).

The layered organisation of the CW changed as the cell matured, presenting a CW with a developed, thick, strongly electron‐dense TLS layer, an underlying, possibly newly formed, and relatively weak electron‐dense inner layer (IL‐d) that thickened during the cell cycle (Figure 1E vs. Figure 1F), and an IL‐nd up to the PM. In addition, further development of the outer TLS was observed with a relatively thin lamellae/laminated structure (TLS in Figure 1F), which was hardly seen in the early stages (Figure 1D,E). The IL‐d further developed with ultrastructural changes, with an outer part adjacent to the TLS containing an electron‐dense layered structure compared to an underlying thick and irregularly electron‐dense inner part. These structural developments to the outer part of the CW during the later stages of the cell cycle appeared to progressively reduce the thickness of the innermost non‐electron‐dense layer (IL‐nd thickness; Figure 1D > E > F), indicating that the CW of mature cells was resistant and tough. In summary, cells in the stationary phase exhibited a thicker CW that was more organised at the ultrastructural level with easily distinguishable layers, collectively contributing to a ca 58% increase in CW thickness (Table 1) compared to young cells.

Intercellular connections of ca 120–160 nm long were observed as extracellular attachments between daughter cells (black arrows in Figure 1C). These structures, possibly with pectic substances, were observed to connect neighbouring daughter cells and thereby likely responsible for keeping cells adhered to each other in the autosporangium. However, during the cellular maturation stage, these structures were not observed, suggesting that there was a degradation process for these substances after the rupture of the MCW and release of the daughter cells.

During the growth phase, we observed more prominent ribs, which became smoother with a somewhat roundish end in more mature stages (Figure 1E vs. Figure 1F). In *Coelastrella* sp. 3–4, the rib width (measured at the middle height of a rib) under control conditions increased from ca 186 to 215 nm during the exponential and stationary phases, respectively (Table 2); however, this increase was not statistically significant (Table 2). The rib height exhibited minimal variation, measuring approximately 220 and 217 nm during the exponential and stationary phases, respectively. Standard deviations were relatively large for all rib measurements, indicating high cell‐to‐cell variability in rib expression (Table 2).

**TABLE 2:** Development of outer cell wall features in the three microalgae across two growth stages under temperature conditions of 25°C (control) and 5°C (cold).

Microalgae strain	Average values ± SD		
	Condition	Exponential	Stationary
*Coelastrella* sp. 3–4 Rib dimensions in nm	Control	Width: 186.38 ± 49.68^a^	215.05 ± 59.26^a^
		Height: 219.69 ± 64.51^a^	216.61 ± 35.79^a^
	Cold	—	—
*Chlorella vulgaris* sp. 13–1 ECM thickness in nm	Control	34.00 ± 9.98^aA^	36.38 ± 13.73^aA^
	Cold	64.92 ± 15.09^aB^	59.40 ± 14.29^aB^
*Scenedesmus* sp. B2‐2 Warts density per cell	Control	40.73 ± 12.12^aA^	59.67 ± 9.66^bA^
	Cold	65.67 ± 15.74^aB^	88.33 ± 8.56^bB^

*Note:* Lowercase letters indicate differences between growth stages for a given strain within a row. Uppercase letters indicate differences between temperature treatments for a given strain within a column. Different letters indicate statistically significant differences (*p* < 0.05). Data are presented as mean values (*n* = 10 cells for ribs and ECM thickness, and for wart counts *n* = 15).

Abbreviations: ECM, extra cellular matrix layer; nm, nanometer; SD, standard deviation.

**FIGURE 2:**
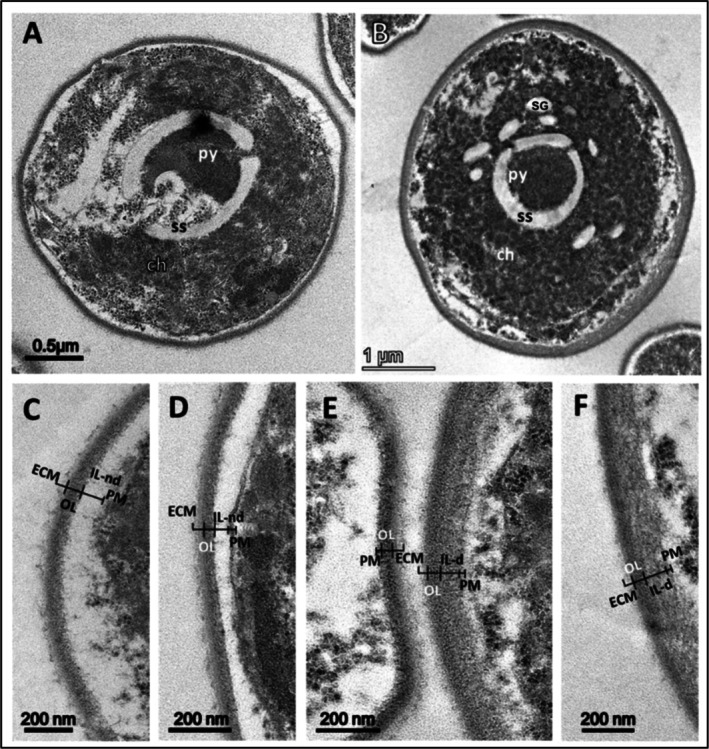
TEM micrographs of *Chlorella vulgaris* sp. 13–1 at 25°C, showing cell wall development during the life cycle starting from daughter (A, C, and D) to vegetative cells (B, E, and F): (A) Longitudinal section of a *C. vulgaris* 13–1 daughter cell; (B) Longitudinal section of a vegetative cell with chloroplast (ch), pyrenoids (py), starch sheath (ss), and starch granules (sg); (C, D, and E (left cell wall)) Cross‐section of daughter cells showing part of the cell walls for fine details of the layered structure; (E (right cell wall) and F) Cross‐section of mature cells illustrating fine details of the cell walls (part). Extracellular matrix = ECM, Outer layer = OL, plasmalemma membrane = PM, inner layer electron‐dense (IL‐d), inner layer non‐electron dense (IL‐nd).

Unicellular microalga *C. vulgaris* 13–1 lacked CW ornamentation/outgrowth and instead displayed a smooth CW (Figure 2A,B) compared to the other microalgal strains of *Coelastrella* and *Scenedesmus* investigated in this study. Regarding CW architecture, *C. vulgaris* did not present a typical TLS‐type CW. Instead, they possessed a bilayer CW in which changes in the organisation were observed throughout the cell cycle (Figure 2C–F). In young *C. vulgaris* cultures, the CWs had a thin electron‐dense outer layer (OL) without discernible sub‐layers and an IL‐nd that extended to the PM (Figure 2 C, D, and left cell in E). In addition, a darkly stained extracellular matrix (ECM) extended over the OL facing the outside environment, and it covered the entire perimeter of the cell but was sparsely dispersed (Figure 2C). As cell development progressed, the CW appeared slightly thicker with the outer electron‐dense layer (i.e., OL) showing further development into an organised layered structure, that is, outermost OL and newly formed adjacent thicker IL‐d layers (Figure 2 right cells in E and F).

As shown in Figure 2E, a major difference between the OL and IL‐d was the packing density of the two layers, where the latter was relatively lighter in density compared to the former. Their development and the resulting distinction were noticeable at the mature stage, when the two layers were visible with the OL showing a much denser structure and the IL‐d possessing a somewhat loose structure reflected by a relatively less electron‐dense region (right cell in Figure 2E vs. Figure 2F). Furthermore, there was a change in the CW structural ECM in which the material density was reduced in mature cells, indicating self‐degradation of the material during cell maturation. For example, the ECM in Figure 2A shows a rough outer surface compared to B, showing a somewhat smooth outer surface with sparsely distributed ECM materials over the OL, which is pronounced in Figure 2C,F. The measured data showed an ECM thickness that remained consistent across growth phases under control conditions (34–36 nm; Table 2).

**FIGURE 3:**
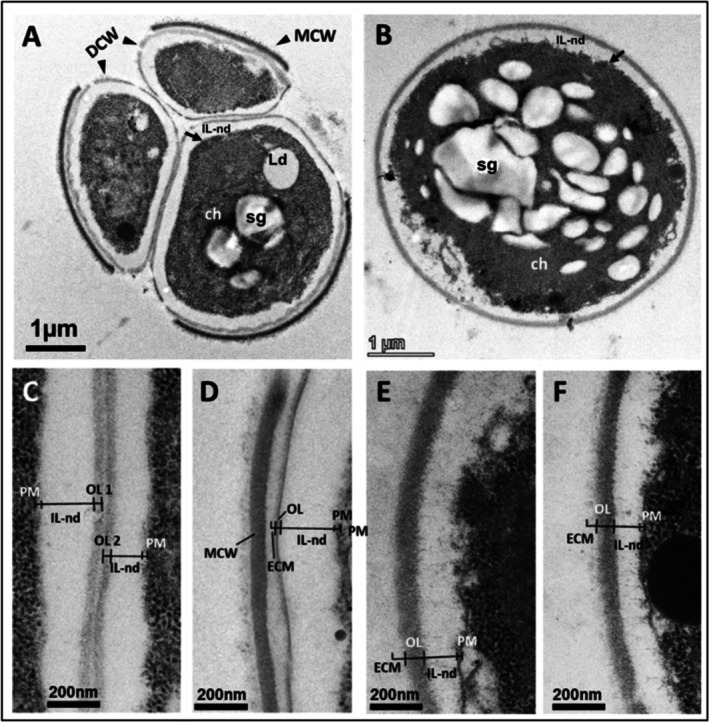
Cell wall (CW) development during the life cycle (daughter (A, C, D) to cells (B, E, F)) of *Chlorella vulgaris* 13–1 grown at 5°C: (A) Longitudinal section of an autosporangium with three young autospores (daughter cells) that are about to release, as indicated by the disrupted mother cell wall (MCW); (B) Longitudinal section of a mature vegetative cell with chloroplast (ch) and starch granules (sg); (C) Cross‐section of two neighbouring daughter cells (part of the CW) showing ultrastructural features and (D) a single daughter cell with a still attached MCW; (E, F) Cross‐section of mature cells showing part of the CW illustrating ultrastructural features. Extracellular matrix = ECM, Outer layer = OL, plasmalemma membrane = PM, inner layer non‐electron dense (IL‐nd) and daughter cell wall = DCW.

*Chlorella vulgaris* underwent a slight morphological modification in response to cold stress (Figure 3). A visible structural change observed during cold stress was the presence of a thick strongly non‐electron dense layer (i.e., off‐white zone, IL‐nd, around the cytoplasm of the cell; Figure 3A,B) between the OL and cytoplasm enveloped by the plasmalemma (black arrows in Figure 3A,B). The thick IL‐nd layer was developed at very early stages of the autospore phase and contained unhatched daughter cells (Figure 3A), possibly shrinking the cytoplasm, as reflected by the dense cellular content. The IL‐nd thickness remained consistent throughout the cell cycle and was visible in mature cells (Figure 3A–F), indicating that this layer was conserved throughout the cell cycle in *Chlorella* even in developed phases under cold stress. In contrast, the OL, which was thin in daughter cells (Figure 3A,C,D), developed into a relatively thicker wall at later stages under cold stress (Figure 3B,E,F). However, the change in CW thickening described above for cells growing under normal growth conditions was likely due to the increase in IL‐d thickness at the mature stages but this layer was not detected in cells growing under low temperature conditions, when the IL‐nd was thick (Figure 2F vs. Figure 3F). This indicates that cold stress replaced IL‐d thickening, which is normal under standard growing conditions, with a newly developed thick IL‐nd layer that was almost undetected in mature cells growing under standard conditions. To the best of our knowledge, this is the first time that this major discrepancy has been revealed in the CW ultrastructure of *C. vulgaris* 13–1 cells growing when comparing control to cold stress conditions. Moreover, cold treatment resulted in ECM thickening during the exponential phase (ca 65 nm), followed by a minor reduction during the stationary phase (ca 59 nm) (Table 2). Cold stress significantly triggered ECM thickening at both the exponential and stationary phases compared to control conditions (Table 2).

**FIGURE 4:**
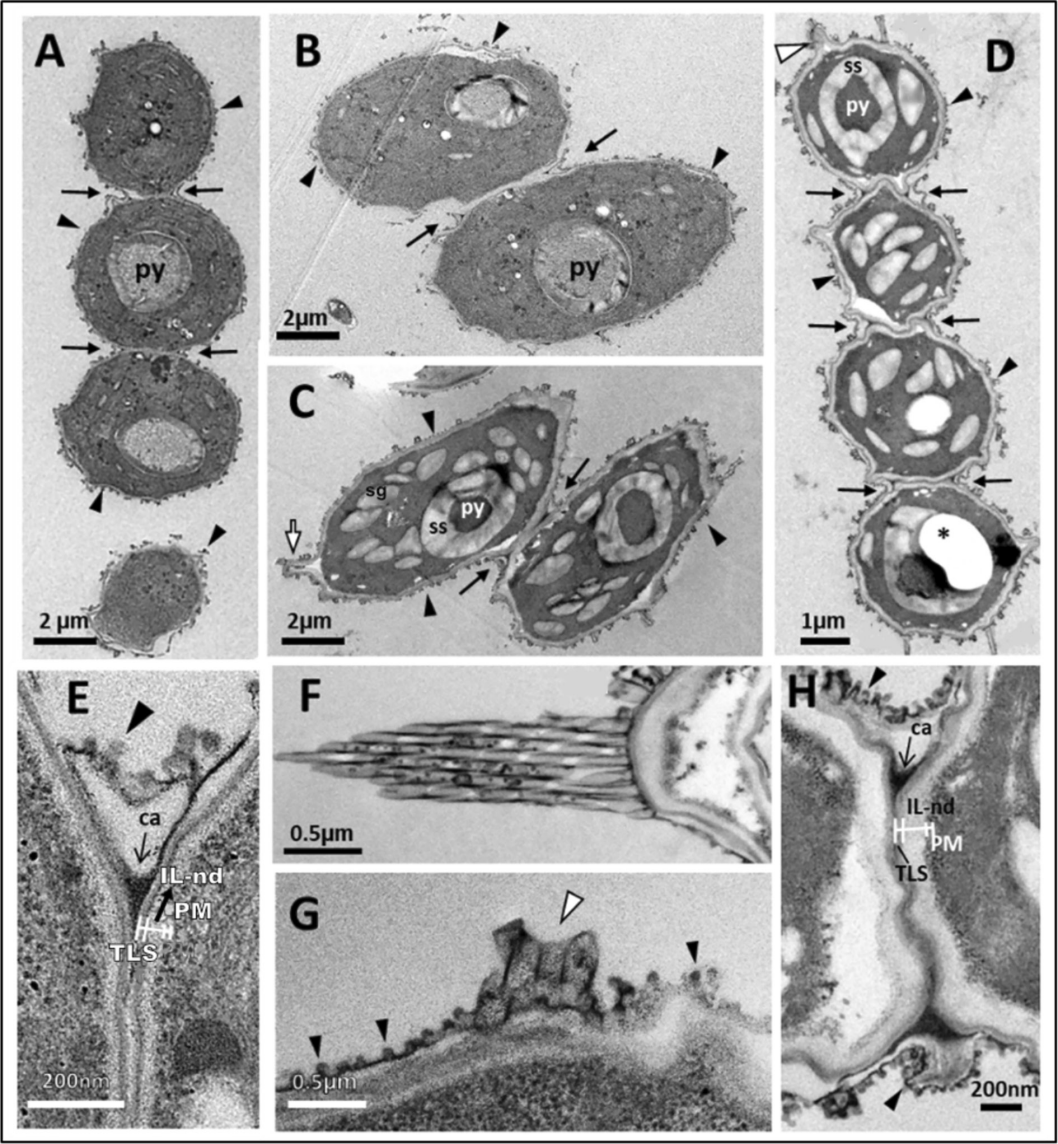
TEM micrographs of *Scenedesmus* sp. B2‐2 under standard conditions at 25°C showing details of cell wall (CW) development and extracellular ornamentation during the life cycle starting from daughter to vegetative cells; (A, B) Transverse (A) and longitudinal (B) sections of young four‐ and two‐cell coenobium; (C,D) Longitudinal (C) and transverse (D) sections of mature vegetative two‐ and four‐cell coenobium; (E) Junction in which 2 young cells of a coenobium were binding to each other, displaying its ultrastructural characteristics, including an electron‐dense warty layer containing warts (arrowhead), plasma membrane (PM), CW, and coenobial adhesive (ca, marked with an arrow); (F) Longitudinal section of a spine showing constituent tubular structures; (G) Longitudinal section of a spine (white arrowhead) attached to the outer surface of warty layer (black arrowheads); (H) Junction with two vegetative coenobium cells illustrating its ultrastructural features, including the warty layer, plasma membrane, CW, and coenobial adhesive. Pyrenoid = py, starch sheath = ss, starch granules (sg), trilaminar sheath = TLS, inner layer non‐electron dense (IL‐nd). Black arrows indicate junctions between cells, black arrowheads for warty layer with warts, white arrowheads for spines and asterisk (*) for artifact of sample preparation during TEM.

Under normal growth conditions, *Scenedesmus* sp. B2‐2 displayed a coenobial cellular organisation mostly consisting of four longitudinally arranged cells, and their CWs having intimate contact with each other (black arrows in Figure 4A,B) and surrounded by a warty‐type layer (black arrowheads in Figure 4). This layer showed varying organisational patterns. During the early stages of cell development, the warty layer appeared to have an orderly arrangement of relatively small warts with some distance between them (black arrowheads in Figure 4A,B). However, during the stationary phase, the layer lost its orderly pattern in cells, and warts became large, prominent, and relatively closely and randomly arranged, creating a rough surface (black arrowheads in Figure 4C,D). Each of the four cells in a coenobium had its own CW underneath the warty layer, which seemed to be somewhat weakly bound to the inner CW, as reflected by its detachment from the CW in some places around the cell, possibly as a result of sample preparation (black arrows in Figure 4A). Rhe warty layer was visible as an outer cover surrounding all four cells and did not play a structural role at connection zones between cells in the coenobium. The connection zones were characterised by darkly stained materials (i.e., black triangular‐shaped regions marked as ‘ca’ with an arrow in Figure 4E) that seemed to bind the outermost parts of the CWs of the two adjacent cells at their first meeting region. Figure 4E shows that the two cells tended to disconnect and separate from each other along the connection zone. However, we did not find any differences in the binding material features between exponential and stationary phases.

Regarding the CW architecture of *Scenedesmus* sp., our observations showed that *Scenedesmus* sp. possessed a CW composed of a TLS‐type OL and a thin IL‐nd that extended to the PM during the early stages (Figure 4E). However, when the cell progressed through more advanced stages, there was a significant increase in CW thickness primarily due to the enlargement of the IL‐nd layer (Figure 4E vs. Figure 4H). The thick IL‐nd layer was apparent when comparing Figure 4A,D. Figure 4D shows an off‐white zone as a layer (i.e., IL‐nd layer) between the outer warty layer and the inner cytoplasm of each cell of the coenobium. In contrast, cells in the exponential phase (Figure 4A) showed no such off‐white zone and thus the warty layer was observed closely attached to the PM/cytoplasm.

Extracellular structures, such as spines, commonly found in *Scenedesmus* spp., were found to co‐localise with the coenobium (white arrows in Figure 4D). These structures were also localised to the terminal cells situated at the apical and basal poles of the cell (white arrows in Figure 4D). Although spines were generally visible as thread‐like single needles protruding from the CW under light microscopy, high‐resolution TEM showed that they had a tubular structure, reflecting nano‐fibrillar arrangements that have a triangular‐shaped appearance in the longitudinal view, as shown in Figure 4F. The spines had a direct association with the TLS‐type outer CW layer, which showed sub‐tubules (i.e., nano‐tubules) at the bottom layer of the spine and direct contact with the TLS layer.

**FIGURE 5:**
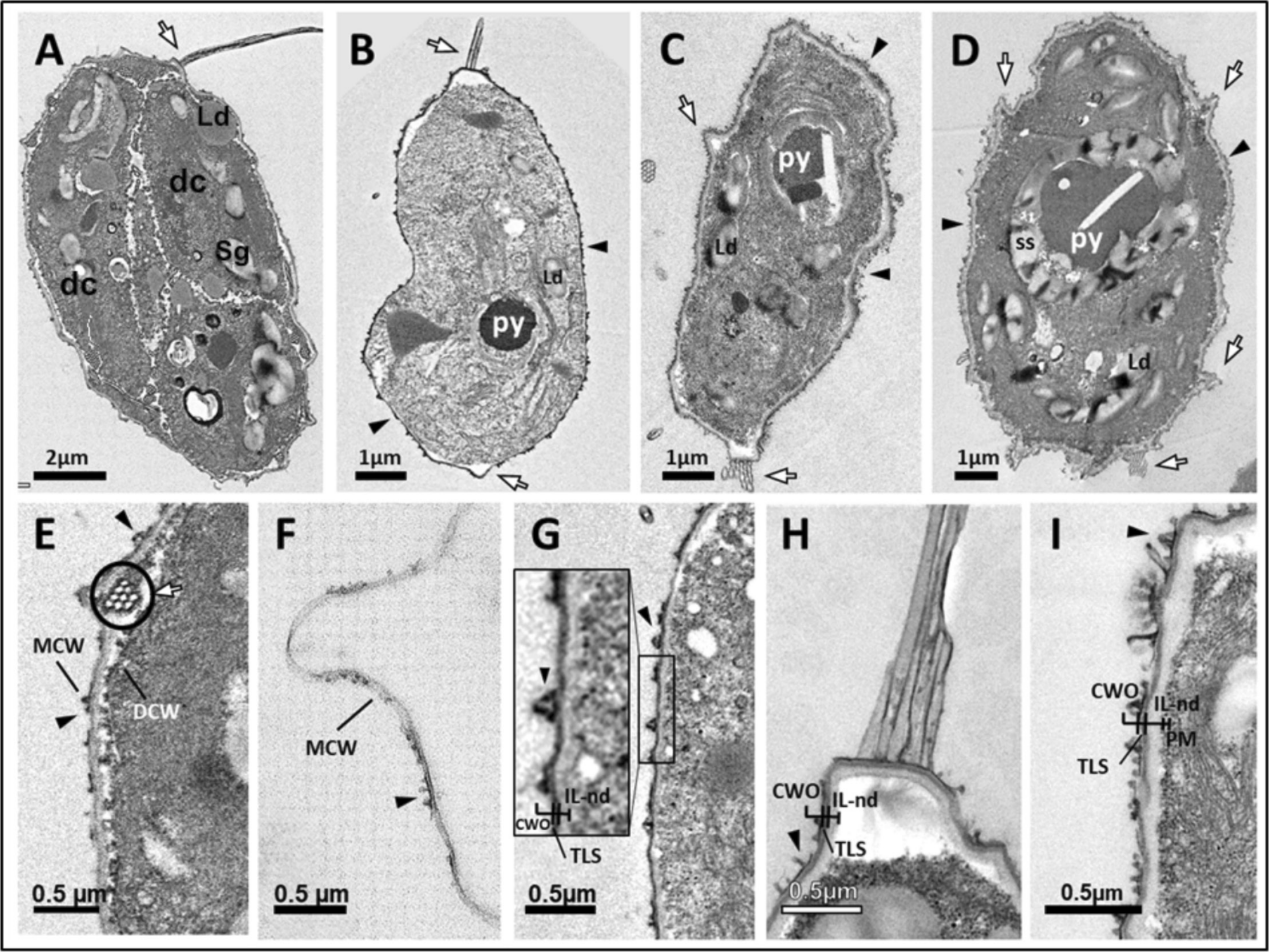
Cell wall (CW) development and details of ornamentation during the life cycle (i.e., (daughter (A, B, E–G) to vegetative cells (C, D, H–I))) of *Scenedesmus* sp. B2‐2 grown under cold stress at 5°C: (A) Longitudinal section of an autosporangium with daughter cells (dc) inside; (B) Daughter cell at an early stage; (C, D) Longitudinal sections of mature cells during the stationary phase; note all cells under cold stress showed differing morphologies, although the basic elliptical/oval shape was retained; (E) Details of the CW ultrastructure of an autosporagium with a spine seen from its cross‐section (black circle and white arrow); (F) Part of the mother cell wall (MCW) released after the hatching process; (G) Part of a CW of a mature cell with ultrastructural details (inset); (H) Longitudinal section of terminal spine showing constituent tubules next to a transverse section of bristle (see black arrowhead); (I) Detailed ultrastructural features of the CW of a mature cell showing different layers. Black arrowheads indicate warty layer details, white arrow for bristles/spines. Pyrenoid = py, starch sheath = ss, lipid droplets = (Ld), Daughter cell wall (DCW), Cell wall ornamentation = CWO, Tri‐laminar sheath = TLS, inner layer non‐electron dense (IL‐nd), plasma membrane = PM.

In contrast, *Scenedesmus* sp. under cold stress (Figure 5) had a predominantly unicellular organisation. Regarding cellular shape, there was variation in the geometry between cells with no consistent morphology observed throughout cell development under stress conditions (Figure 5A–D). They had an elliptical shape outline, but each individual cell showed some deformation along the cell periphery compared to cells growing under normal conditions (Figure 5A–D vs. Figure 4A–D). The darkly stained binding material that was prominent under normal growing conditions was absent under cold stress conditions.

**FIGURE 6:**
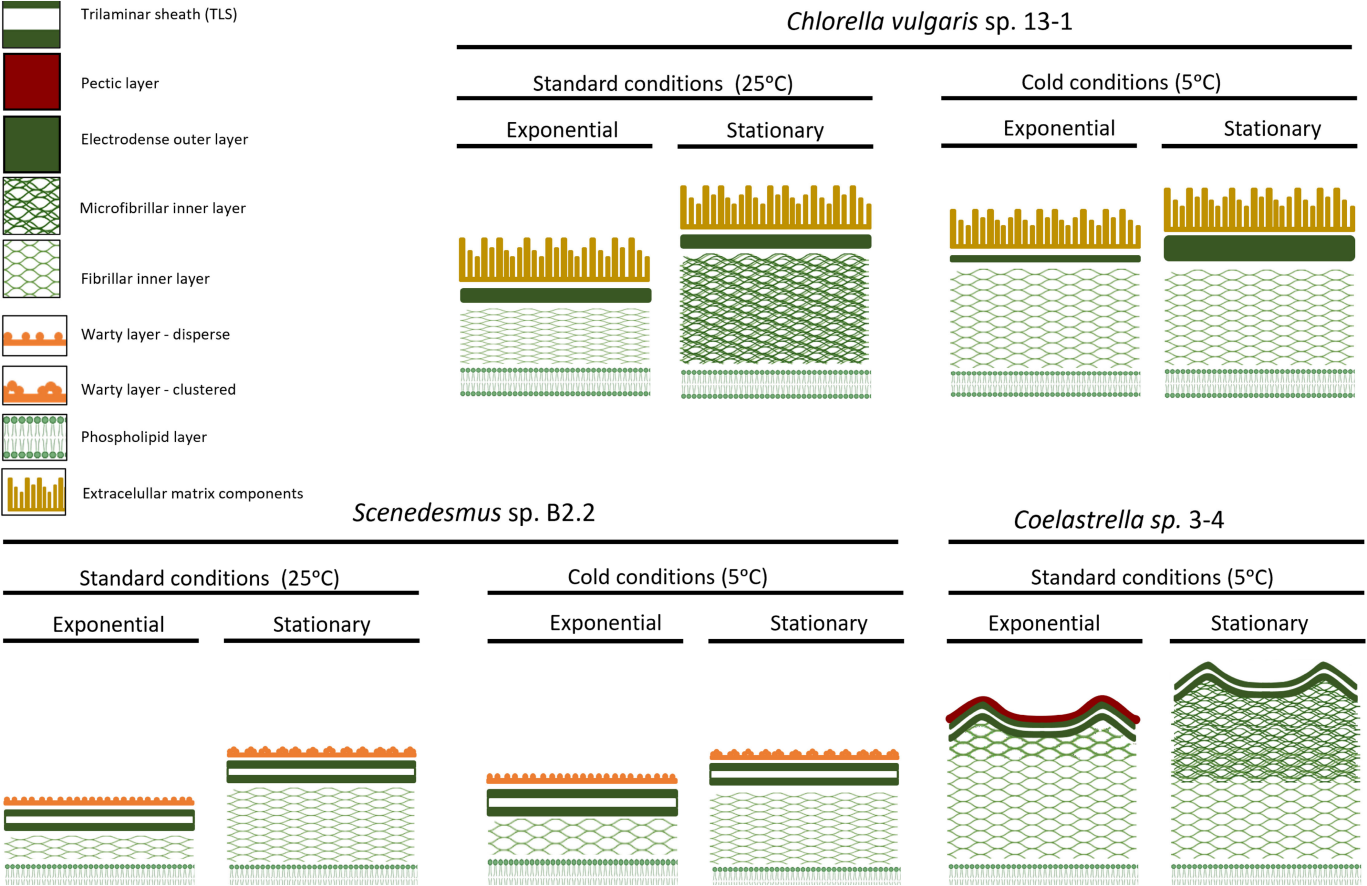
Cell wall structure of three selected microalgae, illustrating the main components and diversity of layer configuration. Layers are not to scale but drawn to be easy to visualise.

Regarding the CW ultrastructure, young *Scenedesmus* cells sometimes showed the presence of MCWs (Figure 5E,F) closely attached to the warty layer and exhibited a thin CW consisting of an electron‐dense TLS OL and a similar in thickness, but not IL‐nd, layer up to the PM (Figure 5G). In addition, the warty layer remained as an outer covering of the cells during cold stress, although the layer with warts was not prominent with less developed warts compared to that observed under normal conditions (black arrowheads in Figure 5). For *Scenedesmus* sp. B2‐2, the number of CW warts was significantly higher under cold stress during both the exponential and stationary phases, increasing from ~41 to ~66 warts per cell perimeter during exponential growth and from ~60 to ~88 during stationary growth (Table 2). As the cell matured, CW thickening occurred primarily due to the increased thickness of the IL‐nd layer but with a similar layered organisation (i.e., warty layer + TLS + IL‐nd + PM) (Figure 5H,I) as observed with cells at 25°C (Figure 4H). The thickened IL‐nd layer surrounding the mature cells grown under cold stress was also apparent in low magnification micrographs, where an off‐white zone was visible between the outer wart layer and inner PM/cytoplasm (Figure 5C,D). Figure 6 shows the CW structure and layer configuration of the three microalgae studied at the exponential and stationary growth phases under two temperature conditions (control at 25°C and cold stress at 5°C).

### 3.3 Variation in the Carbohydrate Residue Composition Between Microalgal Strains and the Effect of Temperature

Table 3 presents the results of glycosyl linkage analysis performed on whole CW samples showing polysaccharide residues in the three microalgal strains (*Scenedesmus* sp. B2‐2, *C. vulgaris* 13–1, and *Coelastrella* sp. 3–4) cultured at two temperatures (25°C and 5°C). Overall, chemical analysis indicated that the CWs of the microalgae exhibited similar linkage profiles with slight changes during low temperature exposure (Table 3). However, *C. vulgaris* presented a profile with more heterogeneous CW carbohydrates, and *Coelastrella* sp. and *Scenedesmus* sp. had more homogeneous profiles, which were preserved during their growth at low temperatures.

**TABLE 3:** Glycosyl linkage analysis of cell wall polysaccharides of *Scenedesmus* sp. B2‐2, *Chlorella vulgaris* 13–1 and *Coelastrella* sp. 3–4 microalgae grown under optimal (25°C)‐ and cold conditions (5°C) at stationary phase.

Linkage	*Scenedesmus* sp. B2‐2		*Chlorella vulgaris* 13–1		*Coelastrella* sp. 3–4	
	25°C	5°C	25°C	5°C	25°C	5°C
	(mol %)	(mol %)	(mol %)	(mol %)	(mol %)	(mol %)
*t‐*Ara*f*	—	—	4.6	3	—	—
*t‐*Ara*p*	—	—	1.1	0.3	—	—
2‐Rhap	—	—	4.5	3.7	—	—
*t‐*Glc*p*	0.4	0.3	5.4	3.5	1.1	0.7
*t‐*Man*p*	2.8	2.7	5.3	4.5	2.4	2.8
3,4‐Rha*p*	—	—	0.3	—	—	—
2,3‐Rha*p*	—	—	3.4	2.1	—	—
3‐Man*p*	2.3	1.3	2.3	4.7	6.9	4.5
4‐Man*p*	20.8	16.4	10	9.6	27.1	22.8
6‐Glc*p*	—	—	0.5	2.7	—	—
4‐Glc*p*	70	75.6	50.5	55.4	58.9	62.8
3,4‐Glc*p*	0.5	0.5	2.6	2.8	0.7	1.7
3,4‐Man*p*	—	—	0.8	0.7	—	—
2,4‐Glc*p*	—	—	—	—	0.6	1.1
4,6‐Glc*p*	3.2	3.2	3.1	3.4	2.3	3.5
3,6‐Gal*p*	—	—	5.3	3.5	—	—

*Note:* ‐, not detected.

In general, 4‐linked glucopyranosyl residue (4‐Glc*p*) was the main component of the CW of all the samples ranging from 50.5% to 75.6% of the total CW polysaccharides at both temperatures. Interestingly, there was a slight increase (ca 5%) in 4‐Glc*p* in all strains under cold stress (Table 3). *Scenedesmus* sp. had the highest percentage (4‐Glc*p*) among the three microalgae, containing 70% and 75.6% under control and cold stress conditions, respectively (Table 3). The second most dominant sugar in the CW of all three strains was 4‐linked mannopyranosyl residue (4‐Man*p*), ranging from 9.6% to 27.1% of the total CW polysaccharides at both temperatures. However, this sugar unit was more abundant in all three strains under control conditions than under cold stress, indicating a similar effect of cold stress with some reduction (Table 3). The highest value of 27.1% was found in *Coelastrella* 3–4 under control conditions, and the lowest value of 9.6% was found in *C. vulgaris* 13–1 under cold stress. Interestingly, 4‐Man*p* in *C. vulgaris* 13–1 varied only marginally between the two growth conditions (Table 3). The features related to the CW composition, cellular and CW organisation, and morphological aspects are summarised in Table 4.

**TABLE 4:** Morphological and biochemical details of the cell wall of the three selected microalgal strains.

Microalgae strain	Temperature (°C)	Cell shape	Cell organization	Cell wall composition		Growth stage	Cell wall features	
				Monosaccharide composition	Glycosyl linkage		Layer organization	Extracellular ornamentation
*Coelastrella* sp. 3–4	25	Spherical	Unicellular	—	—	Exponential	TLS outer‐ and IL‐nd layers	Ribs
	25	Spherical	Unicellular	Glucose, galactose, rhamnose^a^	β‐1,4‐glucan, hemicelluloses	Stationary	TLS outer‐, IL‐d‐ and IL‐nd layers	Ribs
	5	—	—	—	—	Exponential	—	—
	5	—	—	—	β‐1,4‐glucan, hemicelluloses	Stationary	—	—
*C. vulgaris* 13–1	25	Spherical	Unicellular	—	—	Exponential	Single outer‐ and IL‐nd layers	ECM
	25	Spherical	Unicellular	Glucose, galactose, rhamnose^a^	β‐1,4‐glucan, hemicelluloses, arabinans	Stationary	Single outer‐ and IL‐d layers	ECM
	5	Spherical	Unicellular	—	—	Exponential	Single outer‐ and IL‐nd layers	ECM
	5	Spherical	Unicellular	—	β‐1,4‐glucan, hemicelluloses	Stationary	Single outer‐ and **thick IL‐nd** layers	ECM
*Scenedesmus* sp. B2‐2	25	Cylindrical/elliptical	Coenobia (colonies, 2–4 cells)	—	—	Exponential	TLS outer‐ and IL‐nd layer	Warty layer, 4 spines per coenobium, combs
	25	Cylindrical/elliptical	Coenobia (colonies, 2–4 cells)	Glucose, mannose, rhamnose^a^	β‐1,4‐glucan; hemicelluloses, mannans	Stationary	TLS outer‐ and IL‐nd layer	Warty layer, 4 spines per coenobium, combs
	5	Oval/Elliptical (deformed)	Mostly unicellular	—	—	Exponential	TLS outer‐ and IL‐nd layer	Warty layer, multi‐spine per cell, combs
	5	Oval/Elliptical (deformed)	Mostly unicellular	—	β‐1,4‐glucan, hemicelluloses	Stationary	TLS outer‐ and **thick IL‐nd** layer	Warty layer, multi‐spine per cell, combs

^a^ As observed by Spain and Funk 2022. Trilaminar sheath (TLS). Extracellular matrix (ECM). Electron‐dense inner layer (IL‐d). Non‐electron‐dense inner layer (IL‐nd). (‐) No data available. In bold = change due to cold stress compared to normal condition.

## 4 Discussion

### 4.1 Cell Wall Thickness and Architecture During the Cell Cycle and Different Temperatures Using TEM

In the three species studied herein, CW thickness was influenced by the growth phase, in agreement with a previous study (Spain and Funk 2022).

*Coelastrella* 3–4 showed a significantly increased CW thickness during the stationary phase (Table 1).

Young cells displayed a clearly visible layered organization (i.e., TLS + IL‐nd + PM), which was transformed into a much thicker, dense (as reflected by the electron‐dense CW) and structurally distinct layered arrangement in mature cells with a newly developed thick IL‐d layer along with additional structural modifications to the outmost TLS layer (Figure 1B,E,F). A similar layered organisation has also been described in previous studies (Tschaikner et al. 2007; Goecke et al. 2020; Kawasaki et al. 2020).

A distinct characteristic of the *Coelastrella* genus is the presence of extracellular ornamentation in the form of symmetrical meridional ribs arranged from pole to pole (Kawasaki et al. 2020). However, some *Coelastrella* spp. exhibit net‐like rib ornamentation around the CW (Hanagata 2001; Kawasaki et al. 2020) instead of meridional ribs. The thickness of the *Coelastrella* sp. 3–4 CW increased in the area with ribs compared to the area without ribs, in agreement with that reported by Goecke et al. (2020), who observed a similar pattern in mature vegetative cells of *Coelastrella* sp. FGS‐001. The difference in thickness suggests that ribs play a significant role in the overall CW thickness. Similar results were also observed by Kawasaki et al. (2020) in their morphological analysis of *Coelastrella astaxanthina* sp. nov. Ki‐4 vegetative cells. Overall, our results demonstrated differences in the CW thickness of *Coelastrella* sp. depending on the presence/absence of rib zones during the cell cycle. *Coelastrella* rib dimensions showed variability, with large standard deviations suggesting natural heterogeneity in rib formation (Table 2). This reflects the asynchronous development of rib structures within the population, differences in cell orientation during sectioning and imaging, and inherently variable rib morphogenesis in this taxon. Furthermore, the rib width increased more than the height from the exponential to the stationary phase, reflecting a shift in CW remodelling associated with growth cessation and physiological maturation. This preferential lateral thickening resulted in broadened rib bases while the rib height remained relatively stable. This suggests that the increased rib width in *Coelastrella* during the stationary phase represents a form of structural strengthening that enhances wall rigidity and stress tolerance rather than continued outward growth.

TEM revealed the presence of thin, thread‐like extracellular attachments between daughter cells of *Coelastrella* 3–4. The chemical composition of these extracellular connections may be composed of pectic substances, as revealed by histochemical localisation using specific ruthenium red staining for pectins in our recent study (González‐Hourcade et al. 2023). To our knowledge, the nanometer‐sized pectic intercellular connections between daughter cells within the autosporangium of *Coelastrella* sp. have not been described previously, representing a first report of these structures. *Coelastrella* sp. 3–4 did not grow under cold stress; consequently, we do not have data on the CW under cold stress.

The data for *C. vulgaris* 13–1 are in line with previous studies reporting the CW thickness of other *Chlorella* spp. falling within the range of 100–200 nm (Gerken et al. 2013; Sydney et al. 2014). The obtained results suggest that the CW thickness varies among *Chlorella* spp., consistent with the findings of Spain and Funk (2022). Yamamoto et al. (2004) reported an increase in CW thickness of 2–3 nm from the exponential to the stationary phase. In the present study, CW thickness significantly increased from the exponential to the stationary phase, especially under control conditions but even under cold stress (Table 1). Safi et al. (2014) found that the nascent CW of *C. vulgaris* was usually thin and fragile, but the thickness gradually increased during the developing phase. A significant increase in CW thickness was observed in *Parachlorella kessleri*, from 40 to 60 nm from the early to late growth phases (Yamamoto et al. 2005). We observed a slight increase in thickness in MCW thickness at hatching, from 61 nm under control conditions to 77 nm under cold stress (data not shown). The increase in CW thickness was significantly affected by cold stress during the exponential phase compared to control conditions, and this effect was also observed at the stationary phase (Table 1).

In previous studies using other *Chlorella* spp., a variety of layer combinations have been identified. Some species have shown a single microfibrillar layer, while others have a double layer containing outer and inner layers. The outermost cell layer at mature stages likely consists of an electron‐dense CW and a less electron‐dense layer (Yamada and Sakaguchi 1982; Allard and Templier 2000; Gerken et al. 2013). A similar organisation was observed in *C. vulgaris* 13–1 in the present study (Figure 2). Under cold stress, *C. vulgaris* 13–1 showed a major structural change represented by the presence of a thick non‐electron‐dense layer compared to cells grown under control conditions, which showed the presence of a thick electron‐dense layer (Figure 2F vs. Figure 3F). *C. vulgaris* 13–1 exhibited a statistically significant increase in ECM in response to cold stress (Table 2). ECM thickening during the exponential growth phase indicated rapid production of protective extracellular components, whereas a slight but not significant reduction was observed in the stationary phase (Table 2).

In *Scenedesmus* sp. B2‐2, the CW was composed of a TLS‐type OL and a thin IL‐nd extending to the PM, and this type of layered organisation is common in Scenedesmaceae spp. (Bisalputra et al. 1964; Staehelin and Pickett‐Heaps 1975) and confers high resistance to chemical disruptions (Voigt et al. 2014), stress conditions, and predators. *Scenedesmus* sp. B2‐2 showed a significant CW thickening mainly due to cell growth rather than cold stress (Table 1). Using the same strain grown under the same temperature conditions, Spain et al. (2024) observed a CW thickness of 117 nm under control conditions and 156 nm under cold stress during the exponential phase. Consequently, Spain et al. (2024) observed a much thicker CW during the exponential phase than in the present study. However, they recorded a CW increase from control to cold stress of approximately 33%, while the present work showed an increase of approximately 84%. The major difference between the two studies is that Spain et al. (2024) grew the microalgal strain for 10 days, and in the present study, microalgae were grown for 12 and 40 days to reach the exponential phase under control and cold stress conditions, respectively. Moreover, Spain et al. (2024) cultivated the cells under continuous illumination using another cultivation substrate.

The increase in CW thickness was less strong but still statistically significant from the exponential to the stationary phase under cold stress than under normal growth conditions. This may be attributed to the fact that *Scenedesmus* sp. reacted strongly to cold stress during the early growth stage by increasing its CW to a greater extent during the exponential phase (Table 1).

*Scenedesmus* sp. B2‐2 cells were surrounded by a warty layer, which is commonly found in *Scenedesmus* and *Desmodesmus* spp. (Staehelin and Pickett‐Heaps 1975; Shubert et al. 2014). Similar results have also been described in *Scenedesmus pannonicus* by Staehelin and Pickett‐Heaps (1975). *Scenedesmus* displayed a consistent and statistically significant increase in wart density between the exponential and stationary phases under both control and cold stress conditions (Table 2). Moreover, cold stress triggered a significant increase in wart density at both exponential and stationary phases compared to control conditions (Table 2). Cold stress enhanced the CW roughness or rigidity as an adaptive feature of low‐temperature stress.

TEM revealed the presence of connection zones between cells holding them together in a coenobium (Figure 4A–E,H). These connection zones are characterised by an osmiophilic material known as coenobial adhesive (Staehelin and Pickett‐Heaps 1975), which was also observed in the current study (marked as ‘ca’ with an arrow in Figure 4). The material fuses the outermost parts of the CWs of the two adjacent cells, binding them together at this first meeting region. Interestingly, the binding material that was strongly electron‐dense was only observed at the first contact region of the two adjacent cells, existing in a triangular geometry, as shown in Figure 4E (i.e., darkly stained/black triangular‐shaped region), but apparently not located along the connection zone. This indicates that the two cells likely have weak binding along the connection zone, and our recent results on the same species show the presence of minor amounts of pectic substances along this zone, which may be responsible for weakly holding the two cells together along the connection zone (González‐Hourcade et al. 2023). This is evident in Figure 4E, where the poor connection along the zone is apparent as the two cells tended to disconnect along the connection zone. This may have been due to the force exerted during sample preparation, but the two cells were still strongly bound to each other at their first contact region with coenobial adhesive (marked with ‘ca’; Figure 4E). Coenobial adhesive material that was prominent under normal growing conditions was absent under cold stress. This may be related to the change in cell organisation to a unicellular lifestyle, which was a significant deviating feature from the control conditions. Water at 5°C has a higher density than at 25°C, affecting both the physiology and morphology of the cell, especially in *Scenedesmus* strains, which are known for their phenotypic plasticity (Lürling 2003). However, the present study did not identify any differences in coenobial adhesive features between the exponential and stationary phases.

No indication of the presence of propping spikelets was observed in *Scenedesmus* sp. in the present study. These tubular structures are typically found in cells of Scenedesmaceae, between the warty layer and the TLS OL of the CW (Staehelin and Pickett‐Heaps 1975). The strain showed a very high genetic similarity to *Scenedesmus obliquus* (Ferro, Gentili, and Funk 2018), which should not present spines (Bellinger and Sigee 2015; Baudelet et al. 2017). We previously observed spines in *Scenedesmus* sp. when studying the morphological characteristics of the same strain using light microscopy (González‐Hourcade et al. 2023).

In summary, we observed an increase in CW thickness in all three microalgal strains from the exponential to stationary phase. In *C. vulgaris* 13–1 and *Scenedesmus* sp. B2‐2, cold triggered the formation of a thicker CW, especially during the exponential phase. A similar increase in CW thickness has also been observed in vascular plants, in which it contributes to resistance and reduces the risk of CW ruptures during cooling (Huner et al. 1981; Griffith et al. 1985; Stefanowska et al. 1999; Tanino et al. 2013). We speculate that the increase in fatty acids and some soluble sugars, which were previously observed in *C. vulgaris* 13–1 and *Scenedesmus* sp. B2‐2 at low temperatures (Lindberg et al. 2022), together with CW thickening, enables these microalgal strains to tolerate cold stress. The thicker CWs of microalgae can lead to longer downstream processing times. Optimising growth conditions can help control CW thickness and improve efficiency in downstream processing. The results highlight the importance of understanding the CW thickness of microalgae during their growth/cultivation, providing clues for its impact on their processing for industrial use.

### 4.2 Variation in the Carbohydrate Residue Composition Between Microalgal Strains and Effect of Temperature

The CW polysaccharides of microalgae serve as structural constituents of the CW and energy storage and protective polysaccharides and play a role in cell interactions (Moreira et al. 2022). There have been several studies on the CW composition and morphology of microalgal strains (Baudelet et al. 2017; Bernaerts et al. 2018; Colusse et al. 2022; Spain and Funk 2022). The CWs of Charophycean green algae are dynamic with complex structures that comprise networks of cellulose microfibrils tethered by cross‐linking glycans (Sørensen et al. 2011). However, most of these studies have focused on microalgal species of biotechnological interest, limiting work on sub‐arctic species (Baudelet et al. 2017; Colusse et al. 2022).

In addition to 4‐Glc*p* (4‐linked glucopyranose), all three species contained variants of glucopyranose linkages, including 3,4‐Glc*p* and 4,6‐Glc*p* (Table 3). Glycan units are associated with cell surface interactions in algae and coral (Wood‐Charlson et al. 2006). The presence of crystalline cellulose was also evaluated using polarised light microscopy, but none of the microalgal strains or cultures showed evidence of its presence (data not shown). Similar to Glc*p*, mannopyranose units (Man*p*) also existed with other linkage types (Table 3). In *Scenedesmus* and *Coelastrella* grown at a low temperature, the increase in 4‐Glc*p* was followed by a similar quantitative decrease in 4‐Man*p*. Glucose provides rigidity and structural stability to the CW, and mannose provides hydration. Therefore, these two strains increase the glucose level to provide more rigidity and stability to the CW at low temperatures.

In the methylation analysis on the extracted CW of *C. vulgaris* , the presence of a high proportion of glucose and substitutions of mannose, rhamnose, and galactose residues indicated that the sample also contained hemicelluloses, including possible pectin‐type substances. Abo‐Shady et al. (1993) found that the CW of *Chlorella* was composed of 25% hemicellulosic materials. Accordingly, our data support previous findings on the CW characterisation of *C. vulgaris* in which glucose and mannose are the main monosaccharides containing cellulose (β‐1, 4‐glycosidic linkages) and alkali‐soluble hemicellulose (Abo‐Shady et al. 1993; Loos and Meindl 1982; Poulhazan et al. 2021). Relatively higher amounts of t‐Glcp were observed in *C. vulgaris* compared to the other two microalgal strains, suggesting the presence of an amylose‐like polysaccharide. The increase in 4‐Glcp observed in the CWs of the three strains under cold stress likely represents an adaptive strategy for enhancing the structural stability and rigidity of the CW.

Compared to *C. vulgaris* , *Scenedesmus* sp. and *Coelastrella* sp. did not show the presence of arabinose or rhamnose residues. However, as described above, the CW composition profile of the latter two species was similar and relatively homogeneous. This may be related to the fact that both species belong to the Scenedesmaceae family. To our knowledge, this is the first report on the chemical characterisation of *Coelastrella* sp. CWs revealing a complex polysaccharide profile. Spain and Funk (2022) revealed the monosaccharide composition of *Coelastrella* sp. 3–4 and *Scenedesmus* sp. B2‐2, whose CWs contained large proportions of glucose and mannose as the main monosaccharides. Similar results have also been reported by Takeda (1991) and Loos and Meindl (1982). In the present study, we found both species to contain a large proportion of 4‐Glc*p* as well as 4‐Man*p* and 3‐Man*p* residues, with *Coelastrella* sp. having a higher proportion of the latter linkage of mannose residue compared to *Scenedesmus* sp. The presence of terminal mannose was also identified, but its concentration was not altered by exposure to cold stress, suggesting a more constitutive nature as part of the biochemical composition of the CW of *Scenedesmus* sp. and *Coelastrella* sp.

## 5 Conclusion

The detailed investigation of three subarctic microalgal species (*Coelastrella* sp. 3–4, *C. vulgaris* sp. 13–1, and *Scenedesmus* sp. B2‐2) using TEM showed differences in their CW ultrastructure/architecture, including cellular morphology, between growth phases and when grown under cold stress conditions (5°C) compared to control conditions (25°C). All three strains showed an increased CW thickness during the cell cycle, with *Scenedesmus* having the highest increase of ca 230% compared to the other two strains (ca 58% and 44% in *Coelastrella* and *Chlorella*, respectively). The CW ultrastructure of all microalgae showed a distinct layered configuration, and its thickness increase resulted from both the change in the thickness of a certain layer and/or newly formed additional layer(s). Cold stress affected the ultrastructure, ornamentation, and CW thickness of microalgae, with *Scenedesmus* showing the highest effect of a two‐fold increase in thickness during the exponential phase compared to control conditions. Cold stress triggered the development of a novel thick wall layer beneath the outer wall layer of *Chlorella* cells, but *Scenedesmus* showed a different response. The same layered structure observed in *Scenedesmus* cells grown under control conditions remained during low temperature conditions, but they responded to cold stress by increasing the thickness of the inner wall layer. Cold stress significantly modified the cellular morphology and CW ornamentation/outgrowth, such as warts and spines of *Scenedesmus* cells, which transformed their typical four‐cell coenobial cellular organisation to a unicellular organisation. Glycosyl linkage analysis revealed that 4‐Glc*p* was the main component of the CW of all samples ranging from 50.5% to 75.6% of the total CW polysaccharides at both temperatures. Interestingly, all strains showed a slight increase (approximately 5%) in 4‐Glc*p* under cold stress.

## Author Contributions

**María González‐Hourcade:** methodology, formal analysis, investigation, data curation, writing – original draft, visualization. **Dinesh Fernando:** conceptualization, methodology, resources, supervision, writing – review and editing, data processing, funding acquisition. **Francesco G. Gentili:** conceptualization, methodology, resources, supervision, writing – review and editing, data processing, project administration, funding acquisition.

## Funding

This work was supported by Kempe Foundation (JCK‐2008). The authors are grateful for the financial support by Bio4Energy (Targeted Strategic Project No. B4E3‐FM‐2‐02) and EU Interreg Aurora (Project Sustainable Nutrients).

## Data Availability

The data that support the findings of this study are available on request from the corresponding author. The data are not publicly available due to privacy or ethical restrictions.
